# The Impact of Workflow Interruptions, Work Readiness, and Missed Nursing Care on Patient Safety Competency Among New Nurses: A Multicenter Longitudinal Study

**DOI:** 10.1155/jonm/7349427

**Published:** 2026-02-11

**Authors:** Hangna Qiu, Zhengyi Ma, Juntong Jing, Yongkang Fu, Dongrun Liu, Jie Liu, Chaoran Chen

**Affiliations:** ^1^ Nursing Department of Huaihe Hospital, Henan University, Kaifeng, China, henu.edu.cn; ^2^ Institute of Nursing and Health, School of Nursing and Health, Henan University, Kaifeng, China, henu.edu.cn

**Keywords:** missed nursing care, new nurses, patient safety competency, workflow interruptions, work readiness

## Abstract

**Background:**

Patient safety competency is a critical attribute for new nurses. However, limited research has investigated the influencing factors and mechanisms affecting new nurses’ safety competency, indicating the need for further exploration.

**Objective:**

This study aimed to examine the impact of workflow interruptions on new nurses’ patient safety competency and to explore the mediating roles of work readiness and missed nursing care using a longitudinal design.

**Methods:**

From March to September 2024, a three‐wave longitudinal study was conducted among 793 new nurses across 14 hospitals in China using a multicenter, stratified cluster sampling method. A total of 706 valid responses were included. Data collected covered demographic information, workflow interruptions, patient safety competency, work readiness, and missed nursing care. SPSS 27.0 was used for data analysis, and Amos 24.0 was applied to construct a structural equation model to assess the effects and mediation mechanisms.

**Results:**

Workflow interruptions were significantly negatively correlated with patient safety competency (*r* = −0.381, *p* < 0.01) and work readiness (*r* = −0.311, *p* < 0.01) and positively correlated with missed nursing care (*r* = 0.375, *p* < 0.01). Work readiness and missed nursing care acted as sequential mediators in the relationship between workflow interruptions and patient safety competency, accounting for 12.61% of the total effect.

**Conclusion:**

Work readiness and missed nursing care play a chain mediating role between workflow interruptions and new nurses’ patient safety competency. Future interventions should aim to optimize workflow processes, minimize unnecessary interruptions, and enhance new nurses’ training and psychological support to improve their ability to function in complex clinical environments, thereby ensuring care quality and patient safety.

## 1. Introduction

Patient safety competency refers to the knowledge, skills, and attitudes required by healthcare professionals to identify, prevent, and manage risks during the care process, ensuring that patients are protected from harm [1]. These competencies are essential for nurses to provide safe and high‐quality healthcare services and are closely linked to reducing patient harm [2]. Globally, patient safety competency has become a central concern in healthcare systems and a fundamental component of quality nursing care. According to the World Health Organization, approximately one in 10 patients worldwide experiences harm while receiving health care, with more than 3 million deaths annually attributable to unsafe care [3]. In China, the number of reported adverse events has continued to rise. For example, the National Medical Device Adverse Event Monitoring Annual Report documented 694,866 adverse medical device event reports, representing a 6.79% increase from the previous year [4]. Similarly, in 2024, a total of 2.597 million reports of adverse drug reactions/events were recorded, also showing an upward trend year by year [5]. With the advancement of medical technologies and heightened public awareness of patient safety, societal expectations for healthcare institutions and professionals have grown. Global quality management agencies have also incorporated patient safety‐related indicators into performance evaluations to further reduce adverse events [6].

In recent years, numerous countries have examined the patient safety competency of nursing staff. Individual‐level factors, such as personality traits, clinical experience, and professional knowledge, have been identified as key influences [7]. One study found that newly graduated nurses often have lower levels of patient safety knowledge [8], while more experienced nurses with strong clinical judgment are better able to detect potential safety risks. Additionally, inadequate teamwork during interdepartmental collaboration in emergency departments has been shown to impair patient safety [9]. Dutra and Guirardello [10] also found that failure to provide safe care, or poor organizational support for safety practices, threatens overall patient safety. New nurses—those with less than 2 years of clinical experience—are in a transitional phase from student to professional nurse [11]. During this period, they are expected to master complex workflows and clinical protocols within a short time and often assume independent responsibilities within 4–6 months of employment [12], and the mismatch between high workload demands and limited experience poses significant challenges to maintaining patient safety. Given that nurses are in constant contact with patients and responsible for tasks, such as medication administration, condition monitoring, and implementing treatment plans [13], their ability to perceive and respond to patient safety issues is essential. Therefore, identifying the factors that influence patient safety competency in new nurses is critical for shaping effective policy and educational interventions.

## 2. Background

Workflow interruptions refer to task suspensions, delays, or forced task switching caused by internal or external disruptions during work processes [14], and these interruptions are a common stressor for healthcare workers [15]. Studies have shown that nurses may experience between 6.3 and 15.2 interruptions per hour in hospital settings [16], with rates in intensive care units (ICU) reaching up to 40% [17]. These frequent interruptions not only increase work stress but also threaten nurses’ ability to maintain patient safety. For instance, task switching between multiple responsibilities may result in information omission, medication errors, or failure to complete essential care tasks [18]. Westbrook et al. [19] further reported that the increased time pressure and cognitive load caused by interruptions can impair nurses’ decision‐making and response time, ultimately endangering patient safety. Other studies have shown that workflow interruptions are closely associated with increased medical error risks, particularly in high‐stress environments [20]. Prolonged exposure to such interruptions may reduce nurses’ vigilance and capacity to respond in complex situations [21]. Research also suggests that high‐frequency interruptions can lead to missed safety checks or delayed emergency responses due to attentional lapses [9, 22]. These conditions compromise patient outcomes and contribute to reduced care quality. Therefore, we hypothesize that workflow interruptions have a significant negative impact on new nurses’ patient safety competency (Hypothesis 1).

Work readiness refers to the adequacy of an individual’s physical and mental state, cognitive resources, and environmental conditions before task execution [23]. Existing studies have shown that new nurses’ work readiness is influenced by multiple levels of factors, including individual and interpersonal dimensions. At the individual level, Mirza et al. [24] reported that new nurses often encounter challenges, such as limited clinical experience and insufficient critical thinking skills during the transition from education to clinical practice, which weakens their initial readiness upon entering the workforce. Other studies have indicated that new nurses with higher self‐efficacy are more confident in achieving entry‐level competencies, thereby demonstrating stronger readiness to assume clinical roles [25]. At the interpersonal level, Tentama et al. [26] found that inadequate communication skills can undermine new nurses’ performance in team collaboration and handover, while Akçoban et al. [27] suggested that higher levels of ethical judgment or ethical sensitivity facilitate more appropriate clinical responses in complex situations; these factors are closely related to work readiness. In addition, during the early career adaptation of new nurses, system‐level factors form their external work environment. According to organizational socialization theory and the Job Demands–Resources model, new nurses typically experience a “high‐demand, low‐resource” transition period after entering clinical practice. External systems, organizational processes, and cultural environments exert a more substantial contextual influence on their work readiness [28, 29]. However, existing research has paid insufficient attention to the system‐level factor of “workflow interruptions.” In real clinical settings, frequent workflow interruptions may weaken nurses’ psychological and physiological preparedness [30] and reduce their sense of control and professional confidence due to heightened frustration and helplessness. According to the theory of attentional resources [31], individuals’ attentional resources are limited. When nurses continually cope with interruptions in high‐demand situations, their limited resources become further dispersed, resulting in increased cognitive load. For nurses with insufficient work readiness, additional attentional investment is required to adapt to the environment, make decisions, or respond to emergencies, making them more susceptible to cognitive overload. This manifests in reduced information processing efficiency, higher error rates, and diminished safety awareness [32]. Over time, these effects may compromise their patient safety competency and increase the risk of medical errors. Moreover, the ongoing depletion of attentional resources may lead to fatigue, stress, and burnout [33], further impairing nurses’ professional performance and patient safety capability. Therefore, this study hypothesizes that work readiness mediates the relationship between workflow interruptions and patient safety competency (Hypothesis 2).

Missed nursing care is defined as the failure of nurses to provide necessary care as planned or according to patient needs (partial or complete omission), or the delay in the implementation of care in clinical practice [34]. Based on Mcdaniel and Einstein [35]’s prospective memory theory, task interruptions or delays make it easier for individuals to forget or have difficulty resuming the work they were previously engaged in or about to undertake, and reconstructing the memory before the interruption can significantly increase the probability of errors, repetitions, and omissions. This suggests that in clinical work, workflow interruptions may increase the likelihood of nurses forgetting tasks, leading to missed nursing care. Related studies have also confirmed the positive correlation between workflow interruptions and missed nursing care [36]. Ball et al. [37] and Cho et al. [38] argued that when missed nursing care is a predictive factor, the insufficient patient safety competency of nurses may be due to missed care, which in turn increases the incidence of adverse events and patient mortality and reduces the quality of nursing care. In nursing practice, nurses’ patient safety competency includes risk perception, decision‐making ability, and the ability to respond to emergencies. Missed nursing care can weaken nurses’ cognitive resources and decision‐making abilities, thereby affecting their patient safety competency. In high‐pressure nursing environments, nurses need to handle complex nursing tasks with limited time and energy, and missed care may lead to the omission of key safety decisions [39]. Moreover, missed nursing care may weaken nurses’ perception of potential risks, making it difficult for them to identify and respond to patient safety hazards in a timely manner [40]. Therefore, this study hypothesizes that missed nursing care plays a mediating role between workflow interruptions and patient safety competency (Hypothesis 3).

Research also shows that early career nurses’ level of work readiness can influence the quality of their care [41]. For example, a study led by Singapore General Hospital (SGH) in 2024 [42] found that nurses with less than two years of work experience had lower work readiness. About 60% of new nurses felt unconfident when independently performing complex nursing procedures, such as managing emergency clinical situations, tracheostomy care, and chest drainage care. In addition, over 40% of respondents were confused when dealing with ethical issues related to patient care, and about 37% of nurses encountered difficulties when caring for terminally ill patients. These issues may lead to omissions or improper execution of nursing tasks, thereby affecting patient safety and nursing quality. Combining the above discussion of the relationships between variables, this study hypothesizes that workflow interruptions can affect the patient safety competency of new nurses through the chain mediating role of work readiness and missed nursing care (Hypothesis 4).

**Figure 1:**
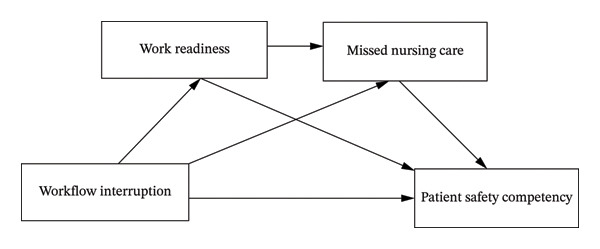
Theoretical framework.

In summary, current research on new nurses’ patient safety competency has not adequately addressed the underlying mechanisms. This study aims to fill that gap by exploring whether work readiness and missed nursing care mediate the relationship between workflow interruptions and patient safety competency, and to construct a comprehensive model of the influencing mechanisms. The theoretical framework is shown in Figure 1.

## 3. Methods

### 3.1. Research Design

This study adopted a multicenter, three‐wave longitudinal design to establish the temporal ordering of variables through a T1 ⟶ T2 ⟶ T3 measurement sequence. The analytical model primarily examined cross‐time lagged associations among the study variables. Work readiness and missed nursing care were measured concurrently at T2 as mediating variables, with the aim of examining their joint effects on patient safety competency within the same developmental stage of newly graduated nurses. In addition, structural equation modeling (SEM) was employed to test the mediating roles of work readiness and missed nursing care. The study was conducted and reported in accordance with the STROBE guidelines to ensure transparency and reporting quality.

### 3.2. Participants

To ensure the representativeness of the sample, a multicenter design was adopted. Participants were recruited from 14 hospitals with varying levels of economic development and healthcare resource allocation. Within each selected hospital, departments were used as units for stratified cluster sampling, and nurses were randomly selected from internal medicine, surgery, obstetrics and gynecology, pediatrics, ICU, and emergency departments. Three departments were chosen from each hospital. Inclusion criteria were as follows: (1) registered nurses with a valid qualification certificate; (2) nurses with less than or equal to 2 years of work experience; and (3) voluntary participation with informed consent. Exclusion criteria were as follows: (1) nurses who resigned or voluntarily withdrew within 2 years of employment; and (2) nurses with prior work experience before employment. Before commencement, approval was obtained from hospital leadership and the nursing departments to ensure ethical compliance throughout the research process.

### 3.3. Data Collection

Data collection was conducted at three time points: March 2024 (T1), June 2024 (T2), and September 2024 (T3). In the first stage (T1), demographic variables and workflow interruptions were assessed among 793 nurses, with 767 valid questionnaires collected. To enhance traceability and rigor, each participant was assigned a unique anonymous ID at T1, which was used throughout T2 and T3 for data matching. Three months later (T2), work readiness and missed nursing care were assessed using the same self‐reported method among valid participants from T1, yielding 735 valid responses after accounting for attrition due to resignation, absence, or incomplete responses. In the third stage (T3, 3 months after T2), nurses who completed T2 were followed up to assess their patient safety competence. After removing invalid responses due to reasons, such as missing key variables, inconsistencies, or unmatched information, 706 valid questionnaires were included in the final analysis. Throughout data collection, logical verification and consistency analysis were rigorously applied to ensure data accuracy and reliability.

### 3.4. Instruments

#### 3.4.1. Workflow Interruptions

Workflow interruptions were measured using the Nursing Workflow Interruptions Scale developed by Yu and Lee [43] and adapted by Chinese scholar Chen et al. [44]. The scale includes 12 items divided into two dimensions: human factors and environmental factors. It uses a 6‐point Likert scale: 6 = at least 5 times daily, 5 = 3‐4 times daily, 4 = 1‐2 times daily, 3 = 3‐4 times weekly, 2 = 1‐2 times weekly, and 1 = rarely. Higher scores indicate more frequent interruptions. The original scale’s Cronbach’s *α* was 0.931. In this study, the overall Cronbach’s *α* coefficient of the scale was 0.914.

#### 3.4.2. Patient Safety Competency

Patient safety competence was measured using the Health Professional Education in Patient Safety Survey (H‐PEPSS), developed by Ginsburg et al. [45] and localized in China in 2016. The scale has 17 items across five dimensions: recognizing and responding to safety incidents, effective communication, safety culture, safety risk management, and human/environmental factors. It uses a five‐point Likert scale: 1 = not confident at all, 2 = somewhat not confident, 3 = unsure, 4 = somewhat confident, and 5 = very confident. The original scale’s Cronbach’s *α* was 0.940. In this study, the overall Cronbach’s *α* coefficient of the scale was 0.949.

#### 3.4.3. Work Readiness

Work readiness was assessed using the New Graduate Nurse Work Readiness Scale, developed by Walker et al. [46] and translated by Chinese scholar Li [47] in 2009. It contains 37 items across five dimensions: social intelligence, work competence, organizational acumen, personal work characteristics, and career development. A 10‐point Likert scale is used, ranging from “strongly disagree” to “strongly agree,” scored 1–10. Higher scores indicate higher work readiness. The original scale demonstrated strong internal consistency, with a Cronbach’s *α* coefficient of 0.920. In this study, the overall Cronbach’s *α* coefficient of the scale was 0.980.

#### 3.4.4. Missed Nursing Care

Missed nursing care was measured using the Miss Care Survey developed by Kalisch and Williams [48] and translated by Si and Qian [49]. The scale consists of 24 items rated on a 5‐point Likert scale from “never missed” to “always missed,” scored 1–5. Higher scores indicate a higher frequency of missed care. The original scale demonstrated strong internal consistency, with a Cronbach’s *α* coefficient of 0.924. In this study, the overall Cronbach’s *α* coefficient of the scale was 0.973.

#### 3.4.5. Ethical Considerations

Although this survey was anonymous and did not involve unethical practices or clinical trials on humans, nor did it pose risks to participants’ physical or mental health, significant efforts were made to address ethical concerns. First, the importance and purpose of the study were communicated to participants, who then voluntarily decided whether to participate. Second, participants were assured that their responses would be used solely for research purposes and that they could withdraw from the study at any time. Finally, the study received approval from the relevant ethics committee (ID number: HUSOM2023‐478).

#### 3.4.6. Data Analysis

All data were analyzed using SPSS 27.0 and Amos 24.0. Descriptive statistics were first used to analyze demographic characteristics and study variables. Pearson’s correlation analysis was used to examine the correlations among workflow interruptions, patient safety competence, work readiness, and missed nursing care. SEM was then used to identify both direct and indirect relationships in the model. The bootstrap method with 5,000 resamples was used to calculate 95% confidence intervals. A *p* value less than 0.05 was considered statistically significant. Model fit was evaluated using the following criteria: *x* ^2^/*d* *f* < 5.0, CFI > 0.90, TLI > 0.90, IFI > 0.90, GFI > 0.90, and RMSEA < 0.08 [50].

## 4. Results

### 4.1. Common Method Bias Test

As all variables in this study were obtained through self‐report measures, Harman’s single‐factor test was conducted to assess the presence of common method bias. The results showed that 11 factors had eigenvalues greater than 1, and the first factor accounted for 39.45% of the total variance, which is below the critical threshold of 40% [51]. Therefore, there is no serious concern regarding common method bias in this study.

### 4.2. Demographic Characteristics of Participants

Among the 706 participating nurses, 75.1% were female and 24.9% were male. A total of 65.9% held a bachelor’s degree. Most participants reported a monthly income of no more than 4000 RMB (45.5%), and more than half of the nurses were employed on a contract basis (53.0%). Additionally, 72.0% of the participants worked in tertiary hospitals, while 28.0% were from secondary or lower‐level hospitals. The departmental distribution of participants was as follows: internal medicine (27.9%), surgery (22.5%), obstetrics and gynecology (8.9%), pediatrics (6.7%), emergency (8.6%), ICU (6.4%), and other departments (19.0%). See Table 1 for details.

**Table 1:** Characteristics of participants (*N* = 706).

Demographics	*N* (%)
Gender	
Male	176 (24.9)
Female	530 (75.1)
Age	
< 30	210 (29.8)
30∼39	383 (54.2)
> 39	113 (16.0)
Record of formal schooling	
Junior college and below	142 (20.1)
Undergraduate degree	465 (65.9)
Master’s degree or above	99 (14.0)
Average monthly income	
≤ 4000	322 (45.5)
4000–7000	273 (38.7)
7000–10000	76 (10.8)
≥ 10001	35 (5.0)
Class of hospital	
Level III hospital	508 (72.0)
Level II and below hospitals	198 (28.0)
Administrative office	
Internal medicine department	197 (27.9)
Surgery department	159 (22.5)
Gynecology and obstetrics	63 (8.9)
Pediatric department	47 (6.7)
Emergency department	61 (8.6)
Intensive care unit	45 (6.4)
Others	134 (19.0)
Labor and personnel relations	
Regular establishment staff	141 (20.0)
Personnel agency	191 (27.0)
Contract worker	374 (53.0)

### 4.3. Correlation Analysis and Significance Testing

Table 2 presents the means, standard deviations, and Pearson’s correlation coefficients among the key variables: workflow interruptions, patient safety competency, work readiness, and missed nursing care. The correlation analysis showed that workflow interruptions were significantly negatively correlated with patient safety competency and work readiness (*r* = −0.381, *p* < 0.01; *r* = −0.311, *p* < 0.01), and significantly positively correlated with missed nursing care (*r* = 0.375, *p* < 0.01); Additionally, missed nursing care was significantly negatively correlated with work readiness and patient safety competency (*r* = −0.565, *p* < 0.01; *r* = −0.555, *p* < 0.01), while work readiness was significantly positively correlated with patient safety competency (*r* = 0.494, *p* < 0.01).

**Table 2:** Descriptive statistics and correlations among the study variables (*N* = 706).

	WI	PSC	WR	MNC	M	SD
WI	1.000				47.62	12.12
PSC	−0.381^∗∗^	1.000			62.73	14.13
WR	−0.311^∗∗^	0.494^∗∗^	1.000		240.40	53.20
MNC	0.375^∗∗^	−0.555^∗∗^	−0.565^∗∗^	1.000	59.77	18.10

Abbreviations: MNC, missed nursing care; PSC, patient safety competency; WI, workflow interruptions; WR, work readiness.

^∗∗^ *p* < 0.01 (two‐tailed).

### 4.4. Mediation Effect Test

**Figure 2:**
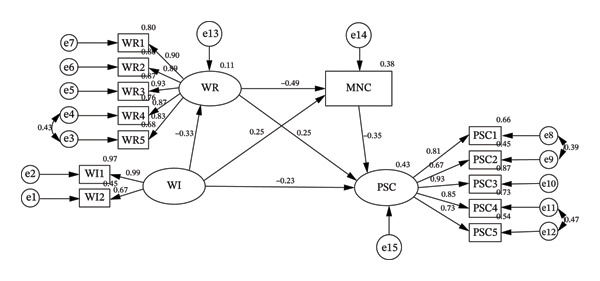
Path analysis diagram of workflow interruption, patient safety competency, work readiness, and missed nursing care. WI, workflow interruptions; PSC, patient safety competency; WR, work readiness; MNC, missed nursing care.

The research model showed good fit indices (*x* ^2^/*d* *f* = 3.979, GFI = 0.954, CFI = 0.977, TLI = 0.969, IFI = 0.977, RMSEA = 0.065). The path diagram and coefficients are shown in Figure 2. The model indicated that workflow interruptions had a negative predictive effect on patient safety competency and work readiness (*β* = −0.33, *p* < 0.01; *β* = −0.23, *p* < 0.01) and a positive predictive effect on missed nursing care (*β* = 0.25, *p* < 0.01); work readiness negatively predicted missed nursing care (*β* = −0.49, *p* < 0.01) and positively predicted patient safety competency (*β* = 0.25, *p* < 0.01). Missed nursing care negatively predicted patient safety competency (*β* = −0.35, *p* < 0.01).

Finally, we tested the mediating effects of work readiness and missed nursing care on the relationship between workflow interruptions and patient safety competency using the bootstrap method with percentile bias correction. The results indicated that both work readiness and missed nursing care have mediating effects, with a total indirect effect of −0.226, accounting for 50.00% of the total effect (−0.452). Specifically, the mediation occurred through three indirect pathways. First, the indirect effect of workflow interruptions on patient safety competency through work readiness was −0.084 (bootstrap 95% CI: −0.121, −0.055). Second, the indirect effect of workflow interruptions on patient safety competence through missed nursing care was −0.085 (bootstrap 95% CI: −0.120, −0.058). Third, the chain mediating effect of workflow interruptions on patient safety competency through work readiness and missed nursing care was −0.057 (bootstrap 95% CI: −0.079, −0.039). Detailed results are presented in Table 3 and Figure 2.

**Table 3:** Bootstrap analysis of the mediating model.

Effects	Paths	Effect	Bootstrap SE	Bootstrapping 95% CI
Total effect	WI ⟶ PSC	−0.452	0.050	−0.543–−0.345

Direct effect	WI ⟶ PSC	−0.227	0.047	−0.320–−0.136

Indirect effect	WI ⟶ WR ⟶ PSC	−0.084	0.017	−0.121–−0.055
	WI ⟶ WNC ⟶ PSC	−0.085	0.016	−0.120–−0.058
	WI ⟶ WR ⟶ MNC ⟶ PSC	−0.057	0.010	−0.079–−0.039

Total indirect effect	Total indirect effect	−0.226	0.024	−0.275–−0.180

Abbreviations: MNC, missed nursing care; PSC, patient safety competency; WI, workflow interruptions; WR, work readiness.

## 5. Discussion

This study primarily explored the impact of workflow interruptions on new nurses’ patient safety competency and examined the mediating roles of work readiness and missed nursing care, with the aim of providing practical guidance for improving nursing quality. The results demonstrated that workflow interruptions have a significant negative effect on patient safety competency, confirming Hypothesis 1. Bertilsson et al. [52] pointed out that accumulated interruptions are a serious source of stress. According to the compensatory control model [53], when individuals face stressors or uncontrollable situations (such as workflow interruptions), they activate cognitive and physiological mechanisms to maintain a sense of order and task performance. In nursing contexts, new nurses often respond to task interruptions by investing additional effort—such as heightened alertness and longer work hours—accompanied by sympathetic nervous system activation (e.g., cardiovascular responses). However, this compensatory mechanism is a “high‐cost strategy”: While it may help maintain short‐term performance, it can lead to energy depletion, attentional lapses, and cognitive overload in the long term [54]. These consequences directly impair critical competencies needed in high‐risk environments, such as recognizing changes in patient condition, making safe decisions, and communicating effectively with the team. From this perspective, the compensatory mechanism provides a more systematic explanation for why new nurses’ patient safety competency is vulnerable under high‐pressure conditions. These findings suggest that nursing managers should pay close attention to how workflow interruptions affect new nurses’ patient safety performance, particularly by optimizing the work environment and task arrangements to minimize frequent disruptions that may undermine their core capabilities. Future studies could further explore intervention strategies based on the compensatory model—such as reducing cognitive load or enhancing emotional regulation support—to strengthen new nurses’ coping capacity under pressure, providing both theoretical and practical guidance for building a safe and efficient nursing practice system.

An important finding of this study is that work readiness mediates the relationship between workflow interruptions and patient safety competency, supporting Hypothesis 2. Previous studies have shown that workflow interruptions can distract nurses from their primary tasks [55], reducing their efficiency and focus. Specifically, frequent interruptions break the continuity of tasks, force constant attention switching, and increase cognitive load, thereby weakening nurses’ sense of control and confidence [56]. This depletion of cognitive resources affects their ability to respond to emergencies and reduces psychological preparedness—ultimately lowering work readiness. Werner and Holden [56] further identified that low environmental familiarity, poor role adaptation, physical fatigue, and inadequate interpersonal communication and collaboration are all hallmarks of poor work readiness, which not only lead to increased turnover intentions among new nurses [57], but also jeopardize patient safety competency—consistent with this study’s findings. Other research suggests that establishing clear communication channels, optimizing task allocation, and minimizing unnecessary interruptions can reduce workflow disruptions [58] and enhance work readiness. Therefore, nurse managers should prioritize workflow continuity and adopt institutional strategies to optimize the work environment, thereby improving both the readiness and safety competency of new nurses.

Another key finding is that missed nursing care mediates the relationship between workflow interruptions and patient safety competency, confirming Hypothesis 3. One of the common consequences of workflow interruptions is errors of omission [59]. A study on factors influencing missed nursing care in Australian public hospitals identified workflow interruptions as a partial cause of medication omissions [60], and this supports the present finding that workflow interruptions disrupt task execution and increase the likelihood of missed care. In turn, nurses who report frequent missed care may receive negative feedback from supervisors or management [61], leading to psychological defense mechanisms and behavioral withdrawal, which diminish focus on nursing tasks and impair their ability to recognize patient risks and make safe decisions—potentially triggering further adverse outcomes. These results underscore the need for nursing management to reduce unscheduled interruptions (e.g., optimize staffing, designate “no‐interruption” periods) to ensure task continuity. Additionally, nonpunitive reporting mechanisms should be established to encourage timely feedback from new nurses. Emotional support and stress management training should also be provided to help new nurses manage negative emotions, enhance focus, and boost safety awareness—ultimately reducing missed care and improving both care quality and safety competency.

Finally, the study revealed that work readiness and missed nursing care serve as a chain‐mediated pathway between workflow interruptions and patient safety competency, supporting Hypothesis 4. Prior studies have indicated that deficits in knowledge and skills, poor role adaptation, communication barriers, and low psychological preparedness can all negatively affect new nurses’ performance and continuity of care [62], contributing indirectly to missed care events. Therefore, nursing managers should implement comprehensive training programs to enhance new nurses’ clinical skills and role adaptation, improve handover and communication processes, and strengthen psychological support to boost mental readiness. In parallel, better resource allocation and workplace conditions are needed to increase overall work readiness, reduce the incidence of missed care, and help maintain care continuity and patient safety competency amid workflow interruptions.

## 6. Limitations

Although this study offers valuable insights for improving the patient safety competency of newly graduated nurses, it has several limitations. First, all data were obtained through self‐reported measures from clinical nurses, which may introduce response bias. Future research could incorporate multiple data collection methods, such as experimental designs, peer‐evaluation questionnaires, and interviews, to enhance the reliability and validity of the findings. Second, the two mediating variables in this study—work readiness and missed nursing care—were measured at the same time point. As a result, the theoretically proposed ordering between these mediators was not directly verified through temporal separation. Future studies may employ cross‐lagged longitudinal designs or experimental approaches to further examine the sequential relationships between these mediators. In addition, although educational level and employment status were not significantly associated with the primary outcome variables in the preliminary univariate analyses and were therefore not included in the mediation model, these factors may still exert potential influences on the outcomes by shaping the mediating processes or exposure pathways, given their theoretical relevance in research on new nurse transition and patient safety. Future research with larger samples or more comprehensive multivariable mediation frameworks is warranted to more systematically investigate the roles of individual characteristics, such as educational level and employment status. Lastly, considering the differences in healthcare systems and challenges faced by new nurses across countries, the findings may not be fully generalizable to other cultural contexts. Therefore, future research should be conducted in diverse cultural settings to examine the cross‐cultural applicability of the conclusions.

## 7. Conclusion

Work readiness and missed nursing care play a chain mediating role in the relationship between workflow interruptions and patient safety competency. This finding suggests that frequent workflow interruptions may reduce the work readiness of newly graduated nurses, thereby increasing the likelihood of missed nursing care, which in turn impairs their patient safety competency. To enhance patient safety, intervention strategies should prioritize optimizing workflow, improving the work readiness of newly graduated nurses, and strengthening the organization and coordination of nursing processes. In particular, enhancing nurses’ sense of task control and efficiency in accessing information can effectively reduce the occurrence of missed nursing care and ultimately improve their safety practices.

## Author Contributions

Hangna Qiu: writing–original draft and methodology. Zhengyi Ma: data curation and formal analysis. Juntong Jing: project administration and resources. Yongkang Fu: writing–review and editing and conceptualization. Dongrun Liu: validation and visualization. Jie Liu: investigation and software. Chaoran Chen: funding acquisition and supervision.

## Funding

The study was funded by the (1) Soft Science Project of Henan Provincial Department of Science and Technology (grant number 242400410523); (2) “Mingde” Network Ideological and Political Studio of Henan University; and (3) Henan Provincial Medical Science and Technology Research Program (RKX202402028).

## Ethics Statement

The study received approval from the relevant ethics committee (ID number: HUSOM2023‐478).

## Consent

The purpose of the study was explained to all participants before the survey was conducted, and informed consent was obtained.

## Conflicts of Interest

The authors declare no conflicts of interest.

## Data Availability

The data that support the findings of this study are available upon request from the corresponding author. The data are not publicly available due to privacy or ethical restrictions.
